# Ethics and biomedical engineering for well-being: a cocreation study of remote services for monitoring and support

**DOI:** 10.1038/s41598-023-39834-8

**Published:** 2023-08-31

**Authors:** A. Maccaro, S. M. Pagliara, M. Zarro, D. Piaggio, F. Abdulsalami, W. Su, M. S. Haleem, L. Pecchia

**Affiliations:** 1 School of Engineering, University of Warwick, Coventry, CV4 7AL UK; 2 Department of Internal Medicine and Medical Therapy, University of Pavia, 27100 Pavia, Italy; 3 Università Campus Bio-Medico, Via Álvaro del Portillo, 21, 00128 Rome, Italy; 4 R&D Blueprint and COVID-19, World Health Organization, Avenue Appia 20, 1202 Geneva, Switzerland; 5 Università di Cagliari, Via Università 40, 09124 Cagliari, Italy

**Keywords:** Quality of life, Biomedical engineering, Lifestyle modification, Psychology, Human behaviour, Information technology

## Abstract

The well-being of students and staff directly affects their output and efficiency. This study presents the results of two focus groups conducted in 2022 within a two-phase project led by the Applied Biomedical and Signal Processing Intelligent e-Health Lab, School of Engineering at the University of Warwick, and British Telecom within “The Connected Campus: University of Warwick case study” program. The first phase, by involving staff and students at the University of Warwick, aimed at collecting preliminary information for the subsequent second phase, about the feasibility of the use of Artificial Intelligence and Internet of Things for well-being support on Campus. The main findings of this first phase are interesting technological suggestions from real users. The users helped in the design of the scenarios and in the selection of the key enabling technologies which they considered as the most relevant, useful and acceptable to support and improve well-being on Campus. These results will inform future services to design and implement technologies for monitoring and supporting well-being, such as hybrid, minimal and even intrusive (implantable) solutions. The user-driven co-design of such services, leveraging the use of wearable devices and Artificial Intelligence deployment will increase their acceptability by the users.

## Introduction

The importance of well-being as a holistic health-related state emerged in 1948 when the World Health Organisation (WHO) expanded the definition of health from a physical state to a psycho-physical one, leading to the bio-psycho-social approach. The term well-being was introduced in opposition to ill-being^1^. Since then, well-being is considered part of the right to health that should be guaranteed to any human being^2^. Furthermore, well-being is enlisted as one of the United Nations 17 Sustainable Development Goals, namely Goal 3 “Good Health and Well-being”, aimed at ensuring healthy lives and promoting well-being for all at all ages^3^.

Being familiar with the WHO’s definitions of well-being and using its significant expertise in the fields of biomedical signal processing, machine learning and eHealth devices, the Applied Biomedical and Signal Processing Intelligent e-Health Lab (ABSPIE Lab) of the School of Engineering, University of Warwick (UoW) were aware of the University of Warwick’s efforts in developing well-being services for staff and students.

Well-being has always been one of the key focus areas of UoW and the ABSPIE Lab. In fact, UoW devised the Warwick Well-being Strategy (2020–2024)^4^ to promote the relevance of well-being among students and staff, and to reflect the breadth of factors that contribute to it. The authors of this paper conducted several studies focusing on mental stress detection using wearable sensors, demonstrating that KETs such as artificial intelligence (AI) and IoT may early detect the onset of mental stress^5–8^. Furthermore, the ABSPIE Lab has extensive experience on the application of IoT and data analytics for remote monitoring of health^9^ and well-being^10,11^. This allowed the Lab to be awarded two important Horizon 2020 research projects, i.e., GATEKEEPER^12^ and ODIN^13^, which are now trailblazers in investigating the application of these KETs for healthcare and well-being monitoring of senior citizens, patients, and healthcare workers.

For these reasons and in conjunction with the University’s “Connected Campus” programme, the ABSPIE Lab designed this multifaceted preliminary study to explore the appropriate introduction of artificial intelligence (AI), internet of things (IoT) and wearable devices leveraging a fast 5G network to support and monitor well-being on the Warwick Campus. The UoW campus is one of the first in the UK to be fully covered by the 5G technology as part of the wider collaborative framework between British Telecom and UoW to foster the benefits of 5G connectivity to accelerate innovation in the West Midlands region^14^. This led to several research and innovation activities with different groups at Warwick University. Our ABSPIE Lab has been involved in a two steps project aiming at involving students and staff in a co-creation study for identifying the scenarios and the KETs that would be more relevant and acceptable to support well-being on campus, while safeguarding individuals’ privacy in respect of the relevant ethical principles, norms, and codes of conduct.

The advantages of an in-situ 5G network with fast, reliable and secure real time data transfer allied to ABSPIE ehealth expertise highlighted the possibility that healthcare and health promotion services on university campuses could be enhanced by deploying disruptive technology to support well-being services, in particular to young students^15^, who have been experiencing new educational and existential challenges (e.g., COVID-19 isolation).

Donato et al.^16,17^ affirm that well-being directly affects student and staff outcomes. The psychologist Seligman describes well-being as a state of overall mental and physical health, strength, resilience, and fitness to function well at work and outside of work. Seligman goes on to state that well-being is in contrast with the concept of happiness that is a transient, short-term emotion, which cannot be sustained for long^18^.

The use of key enabling technologies (KETs) such as IoT, 5G, wearable sensors, data analytics in large organizations (e.g., universities and other large employers) can support well-being as well as help redesign their services, facilities and risk assessment and management procedures. In fact, these KETs can be used for monitoring in real time relevant and appropriate information (e.g., air quality, the number of room occupiers vs the maximum allowed room capacity), supporting early risk detection, and facilitating the actuation of mitigation plans. After all, in the digital age of the twenty-first century, i.e., the condition of living in a digital culture, is deeply intertwined with everyone’s life.

This paper presents the results of the first part of the aforementioned project where students and staff members were invited to participate in focus groups aiming at eliciting their needs and requirements, as an important input to the design, development, and implementation of future solutions for well-being monitoring and support. The second part of this study will accommodate the input from the focus groups for study protocol design involving deployment of the key enabling technologies for data acquisition related to real-time holistic well-being monitoring.

It is hoped that this multifaceted project will pave the way for future projects but on a wider scale and in different settings.

## Methods

**Figure 1:**
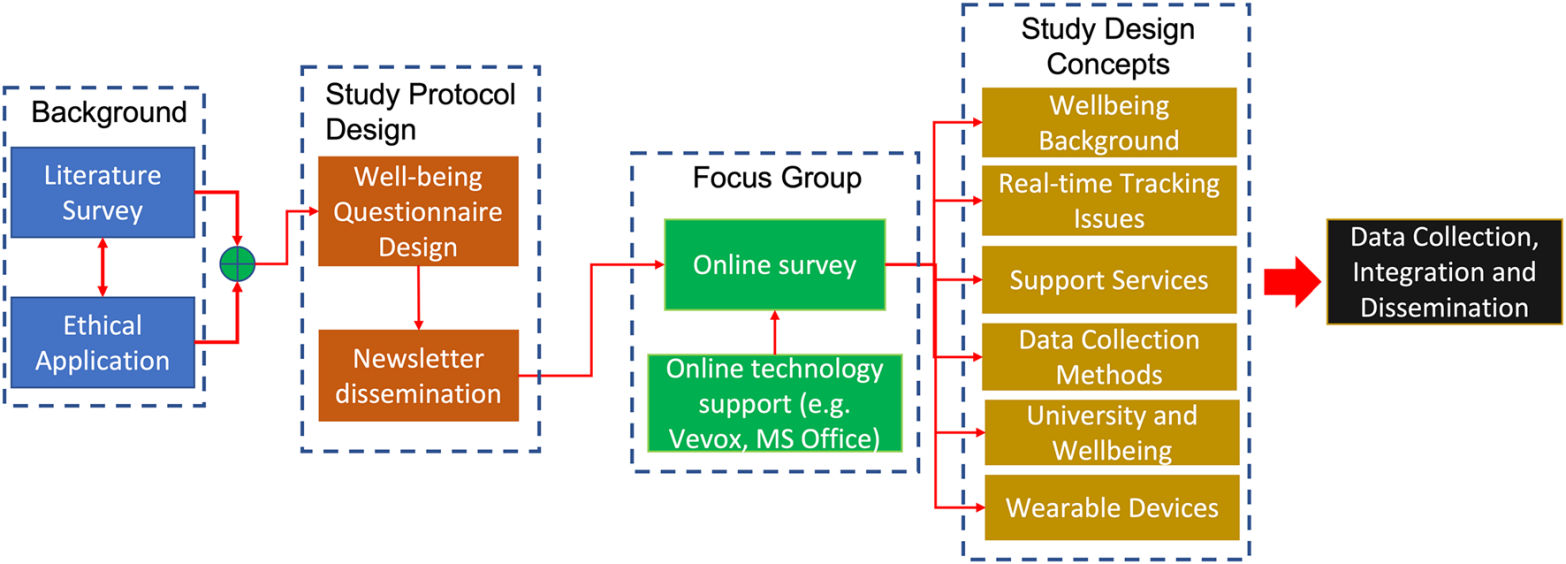
Block diagram of focus group methodology.

This paper presents the result of the first phase, i.e., the focus groups. The focus groups were moderated by a postgraduate student involved in this research project. This was done to follow a peer-to-peer strategy that would allow the students involved not to feel embarrassed and to be in a comfortable situation, being interviewed by their peer^19^. Likewise, the staff involved might be more at ease discussing issues with a designated student as opposed to a colleague or faculty member. This paper presents the distribution of results in bar charts or pie charts because the focus groups are assessed through Likert scales, not allowing a statistical analysis, as there is no average between *“Strongly agree”* and *“Disagree”*. As previously mentioned, this project was divided into distinct phases (Fig. 1):

1. Background. This phase entailed different sessions within the research group, the Warwick Well-being services and their experts, a grey literature review, and the preparation of the Ethical Application for conducting a focus group. The main purpose of this phase was to develop the concepts around well-being, how it can be tracked, current well-being support services, involvement of AI and KETs to improve real-time tracking and issues associated with these technologies. This was followed by co-creating a study protocol to explore the use of AI and IoT for remote real-time well-being support. This study was carried out in accordance with relevant guidelines and regulations, and it got the approval by the Biomedical and Scientifical Research Ethics Committee (BSREC) of UoW (BSREC146/20–21).
2. Study Protocol Design. This phase involved (i) Well-being Questionnaire design and (ii) Dissemination. The well-being questionnaire was agreed and designed with the support of distinguished experts in the field of psychology, led by Prof. Dr. Nincole Tang, member of the Warwick Centre for Mental Health and Well-being Research Centre. It has been based on the state of the art of the current well-being services, the currently outstanding issues, and the involvement of AI and IoT to address these issues. This design process was followed by the preparation of the advertisement material to attract students and staff participants to the focus group. This advertisement was shared on the ABSPIE Lab webpage, and with the support of Students Union/Associations, via engineering and university newsletters. Each and every participant, after reading the leaflet were offered the possibility to withdraw data if they felt uncomfortable, participants completed and signed an informed consent, sent via email, before the focus group attendance. The study was advertised to School of Engineering students and staff because basic competence in understanding device design was needed to allow the proposal of possible technological solutions. Furthermore, the specific decision of involving the students (Generation Z) is for their technological and digital skills^20^.
3. Focus Group. The focus group study involved the dissemination of questionnaires. Online technologies such as Vevox, MS PowerPoint etc. were used. The focus group, because of the COVID-19 restrictions, was conducted on Microsoft Teams with the help of Microsoft PowerPoint and Vevox for real time polling (e.g., using multiple choice or text responses). Some questions were followed by an open floor discussion, prompted by the moderator. This generated well-being study design concepts that fell into the following macro areas:
  - The concept of and thoughts of well-being
  - Well-being tracking; duration, concerns and reasons
  - Evaluating well-being monitoring and support services
  - Well-being tracking: data collection, methods, frequency, and analysis
  - University and your well-being
  - Wearable devices
4. The data management phase. As a preliminary step, the ABSPIE Lab Team shared a data repository based on impact of stress on vital signs^7^ to perform data analytics as described in the study protocol.

### Transparency and openness statement

In this research report we describe how we determined our sample size, all data exclusions, all inclusion/exclusion criteria, whether inclusion/exclusion criteria were established prior to data analysis, all manipulations, and all measures in the study.

The conditions of our ethics approval do not permit public archiving of anonymised study data. Readers seeking access to the data should contact the lead author. Access will be granted to named individuals in accordance with ethical procedures governing the reuse of data, including completion of a formal data sharing agreement and approval of the local ethics committee.

## Results

The focus group sessions took place over two sessions, on Monday 7th March 2022 and Monday 21st of March 2022 at 3:00 PM, each lasting approximately 90 min. The focus group sessions were mixed cohorts with a total of 19 participants (63% male and 27% female). The sample size was determined by the saturation threshold, i.e., the number of focus groups were stopped when no new codes or themes emerged from the interviewees, rather, the same ones started recurring^21,22^. Furthermore, as suggested by experts, qualitative research methodology focus groups are generally not large, 8–12 people^23^. All participants were members of the UoW as either staff or student and completed a consent form prior to the session, only collecting information on age, gender, and email address. The mean age was 28.6 years with a standard deviation of 10.3 years. Ratio of student to staff (SSR) was 3.75:1, which is an abundant ratio given the SSR is much higher at 16:1 as analysed by the University College Union. This means for the demographic of student and staff, there was an adequate and representative selection range used. The following sections report the results organized accordingly to the macro areas mentioned above.

### The concept of and thoughts of well-being

**Figure 2:**
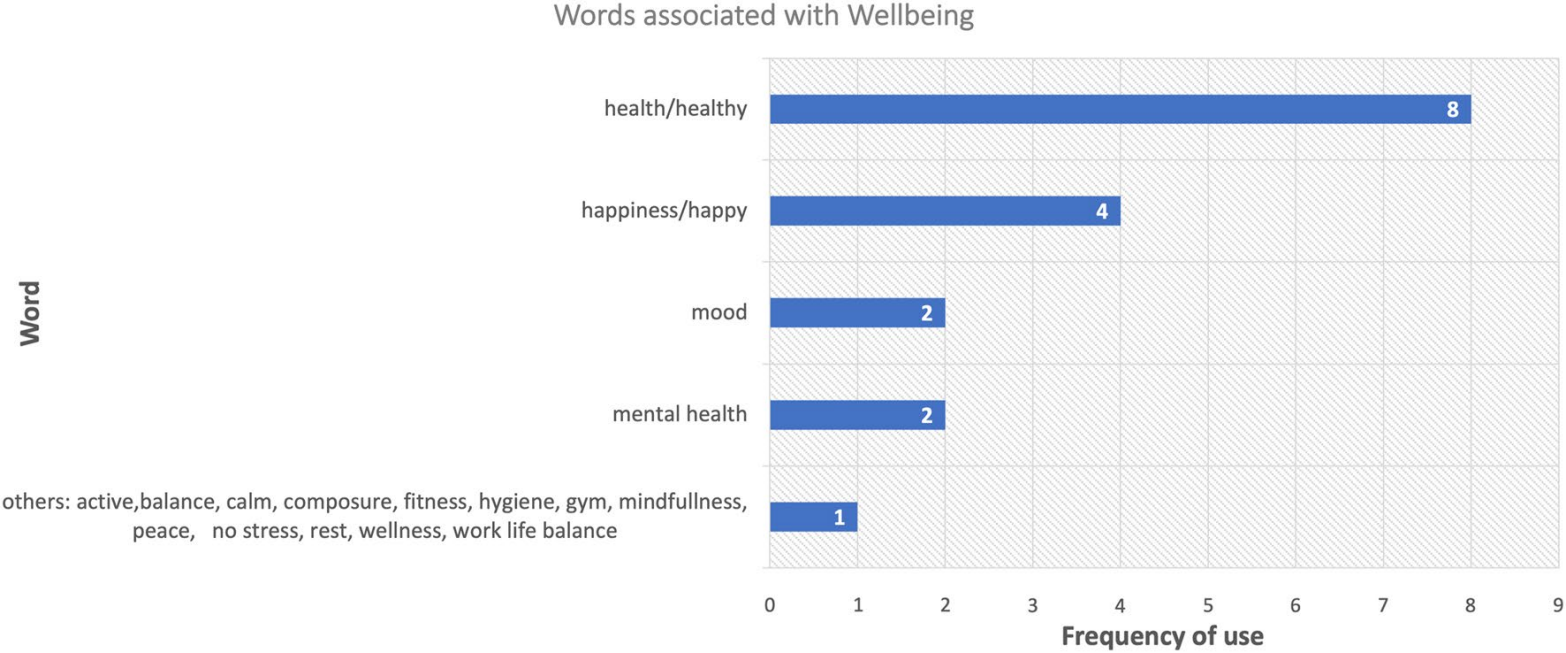
Response to “what words come to mind when you think of well-being?” Sum is greater than total number of participants as each participant could provide more than one entry.

Figure 2 shows the results of the first question used to establish participants’ concept of well-being.

**Figure 3:**
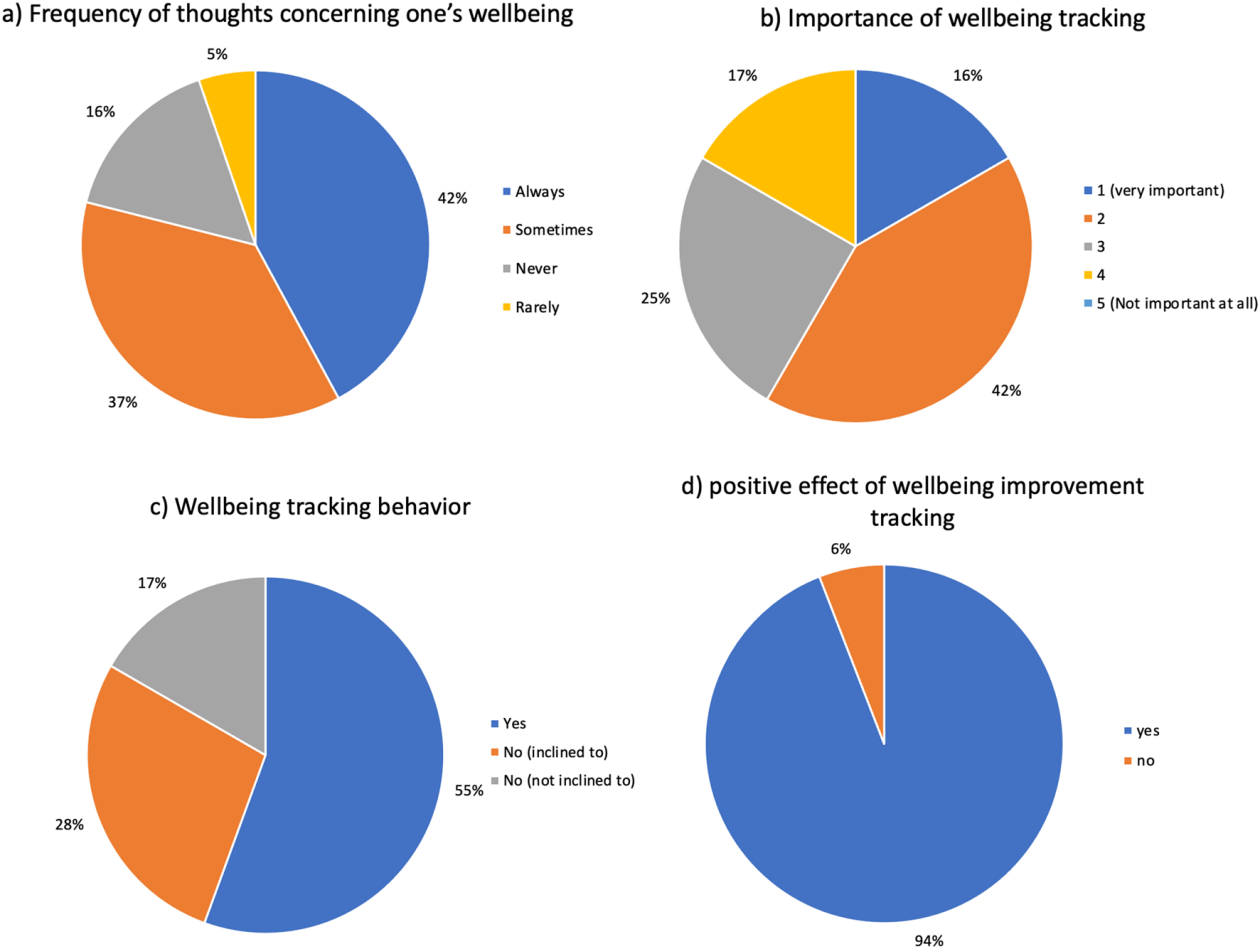
(**a**–**d**) Pie chart representation of poll results of interview questions delivered during the focus group in the first section. Question asked: (**a**) How often do you think about your well-being? (**b**) How important How important is it for you to be able to track and improve your well-being? (**c**) Do you currently track your well-being? (**d**) Do you think that monitoring your well-being can allow you to improve it?

Most participants thought of their well-being frequently and as an important aspect to focus on as they recognised its effects on their work/studies. Most participants use methods to track their well-being and they believed tracking their own would lead to an overall improvement. Figure 3 shows the answers distribution in a pie chart because statistical analysis is not applicable in Likert scale method.

### Well-being tracking; duration, concerns and reasons

**Figure 4:**
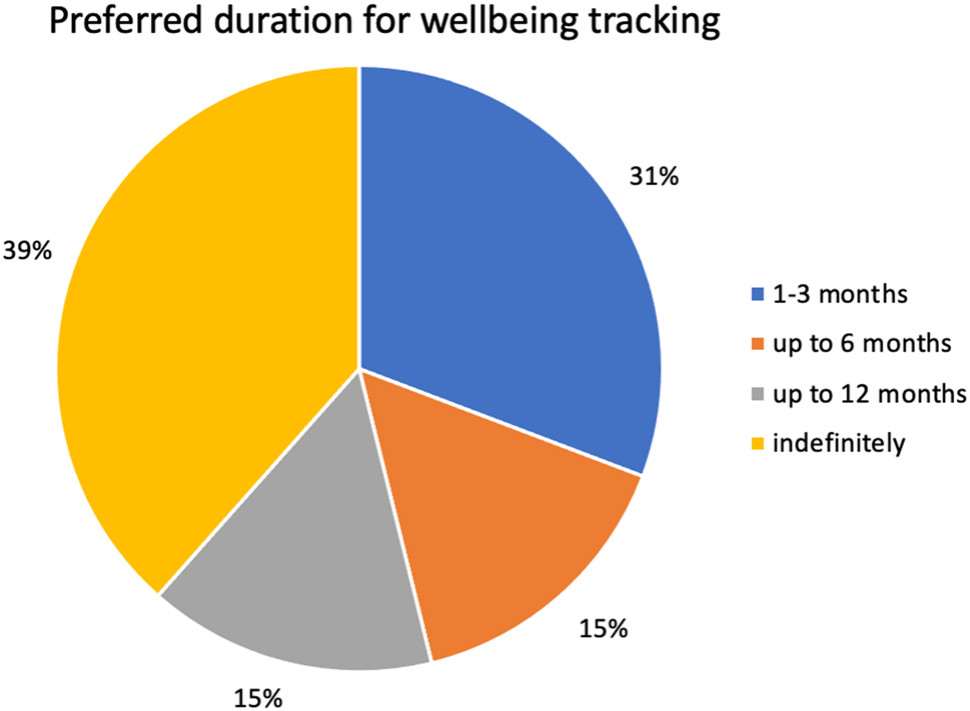
Pie chart representation of poll results of preferred duration of well-being tracking. Question asked: Over how long would you be interested in tracking your well-being?

The results showed that participants would like to track their well-being consistently and long-term (see Fig. 4).

The outcomes of the discussion showed that people monitored their well-being to identify trends to help them avoid taking activities that would produce undesirable results and to keep a logbook of their experiences so they could learn from them in the future. The discussion revealed that, although people thought well-being was important, improving well-being itself should be the focus and not just tracking it. Other insightful remarks included maintaining a sense of self and understanding how it is evolving over time.

In the discussion, they shared the methods they currently use, at same time expressing a lack of awareness on how to consistently use them towards a real well-being.

The reported methods to track their well-being are the following:

1. Specific devices such as a smart watch.
2. Software or device applications. Such as the “Better points” app by Warwick.
3. Manual or digital notetaking for personal well-being tracking to reflect on one's own actions.
4. Journaling—writing about actions during or at the end of the day.

The identified advantages and disadvantages to well-being tracking provided by the participants are provided in Table 1.

**Table 1:** Advantages and disadvantages of well-being tracking and support provided by the participants.

Advantages	Disadvantages
Identifying triggers	Source of stress
Positive influence of behaviour towards desired outcome	Subliminal persuasion
	Distraction

Nevertheless, participants confirmed that they would like to track their well-being and receive support.

### Evaluating well-being monitoring and support services

**Figure 5:**
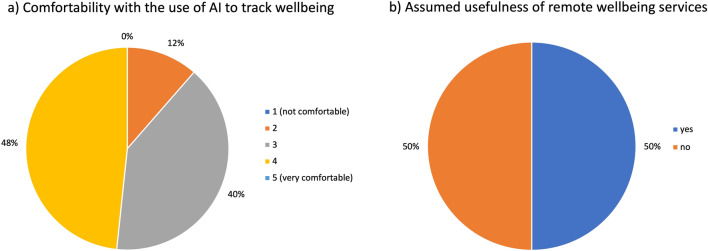
(**a**,**b** Pie chart representation of poll results of interview questions delivered during the focus group in the third section. Questions asked: (**a**) How comfortable are you with the use of artificial intelligence to monitor your well-being? and (**b**) Do you think remote services for monitoring and supporting well-being are useful?

The findings demonstrate that while most participants were at ease with the use of AI to measure well-being, certain concerns were voiced. The main issues were the inaccuracy of interpolations when data was scarce, the inability to decide which data and context were appropriate for assessing well-being, the uncertainty surrounding the method of incorporating AI into assessing well-being, and the lack of "multi-level experience", which participants thought was essential for a thorough well-being examination (see Fig. 5).

### Well-being tracking: data collection, methods, frequency, and analysis

**Figure 6:**
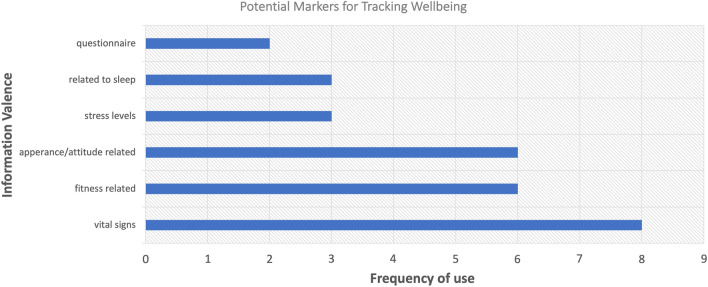
Bar chart representing the valences suggested as markers to track well-being.

Most of the participants identified stress level and heart rate as meaningful well-being indicators, as well as sleep quality and weight fluctuation as shown in Fig. 6.

All participants preferred the well-being tracker to rely on both user-input and automatically measured data (e.g., using sensors), unless they have not to choose one only.

**Figure 7:**
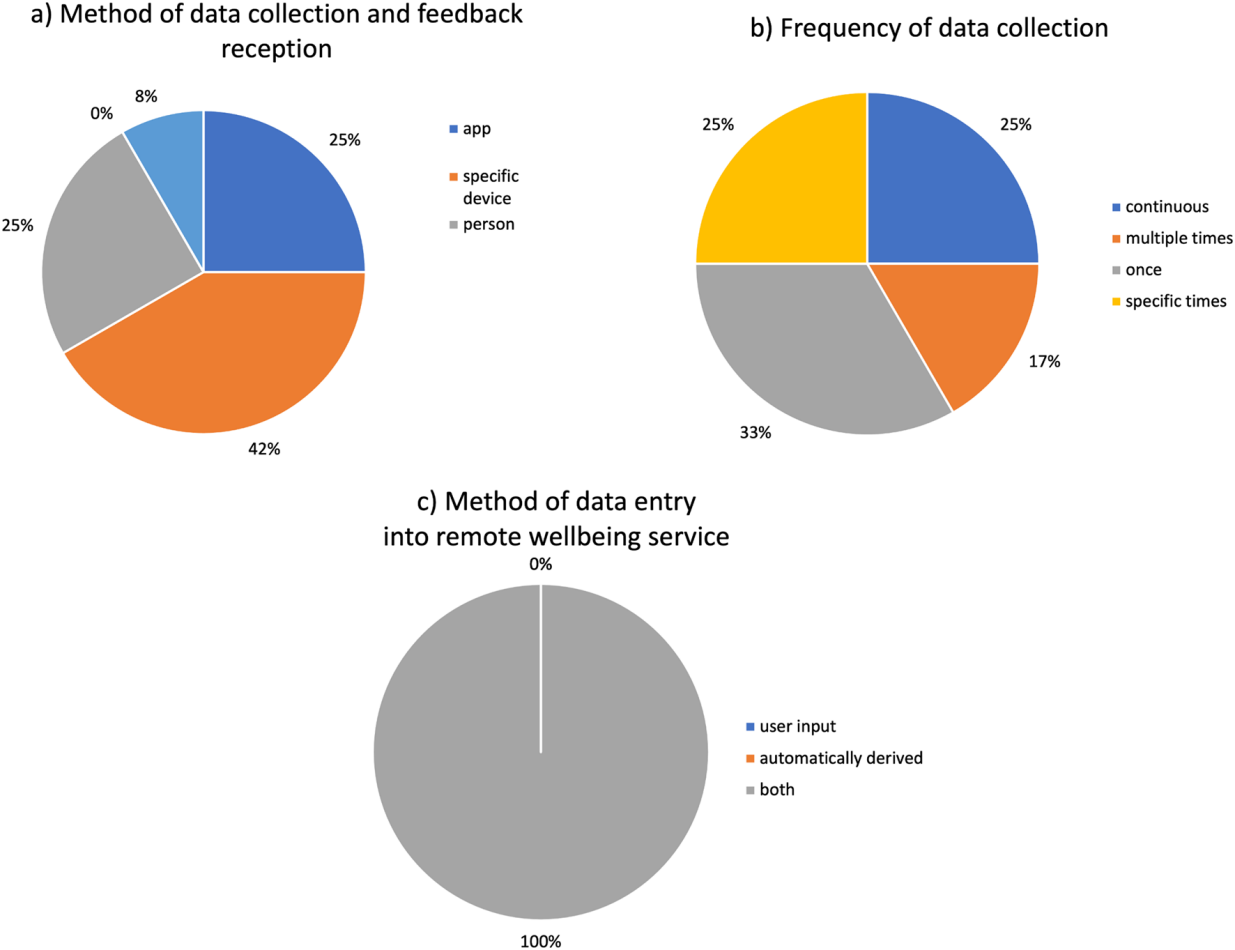
Pie chart representation of poll results of interview questions delivered during the focus group in the fourth section. Questions asked: (**a**) How would you like information about your well-being to be collected? (**b**) How often would you like the required information to be tracked and collected? and (**c**) Would you prefer the well-being tracker to rely on user-input or automatically derived (e.g., using sensors)?

The poll results (Fig. 7a) and discussion revealed that most people were comfortable with a variety of methods for data collection and information delivery, with importance placed on ability to provide holistic and accurate information about well-being. There is an overlap among these methods, and it was agreed that the joint use of such methods linked to the same database for information collection would be preferred for flexibility.

Figure 7b shows the results for the frequency of data collection. There was consensus about the fact that user interactions with the app should be lower, but automatic data collection, e.g., physical activity and step numbers, can be continuous. The interviews (Fig. 7c) also revealed a majority preference of automatic data gathering versus a manual user-input (Supplementary material 1, Supplementary material 2).

The dialogue showed which markers the participants consider helpful in understanding one's well-being (shown in Fig. 6), along with recommendations for how the measurements may be taken (shown in Table 2):

**Table 2:** Summary of the features and forms of measurement to track well-being as suggested by participants, listed in order of importance ascribed by the participants (also visually presented in the word cloud in the supplementary material).

Information valence	Type	Measurement method
Heart rate	Quantitative	Via a device, either ring or watch
		Skin galvanic response as a secondary measure of stress
Individual stress scores	Qualitative/quantitative	Individuals score stress levels based on a uniform scale but scoring system individual to each user
Habit tracker	Qualitative/quantitative	

### University and your well-being

**Figure 8:**
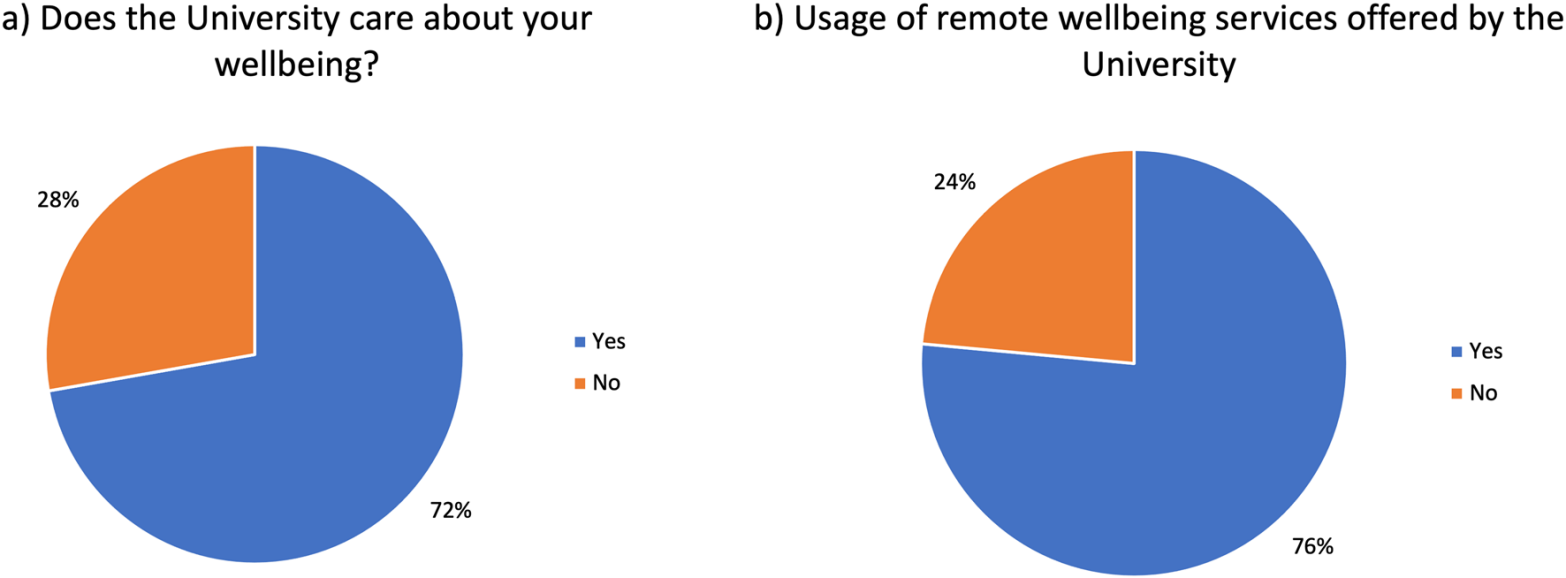
Pie chart representation of poll results of interview questions delivered during the focus group in the fifth section. Question asked: (**a**) Do you think the University cares about your well-being? and (**b**) Would you be comfortable in the university tracking your well-being using remote services?

The results revealed that most participants believed Universities, including the University of Warwick, care about well-being (Fig. 8b). This is because Warwick Well-being offers services like Warwick Nightline and other initiatives which mention well-being, offer free counselling services that would otherwise be expensive, drop-in sessions (up to two hours) for in person “relaxing” activities, such as dog walking, university counselling services offered both in person and remotely, online modules to improve well-being with worksheets and activities, and the Better Points application (in partnership with University of Warwick) (Better Points 202,224). Although most participants knew about and used this service, a substantial number, 24% were unaware of these services in place (shown in Fig. 8a).

Data protection is the main reported concern related to a well-being tracking by the University, i.e., the anonymity of information collected. The discussion revealed an even split between those who preferred anonymized and personalized tracked information, Table 3 summarises the points raised.

**Table 3:** Summary of the arguments postulated for and against preserving anonymity of data collected from well-being tracker).

	Reason	Explanation
Arguments for anonymisation data	To keep the university in the loop	To better understand the specific situations and circumstances of students
	To quicken the university’s response time	It would be useful if information about well-being is shared with the university, it can facilitate allocation of support systems such as auto referral for counselling, or deadline extensions without the need for mitigation forms, etc.
Arguments against anonymising data	Fraudulent information	For example, used to escape from their own duties (i.e., people using sick days when not actually sick)
	Possible infringement of data rights	The tracking instruments should follow existing laws and regulations, such as GDPR (General Data Protection Regulation) as it collects personal and confidential information
	Targeted scrutiny	Might be used against single individuals (i.e., to penalize or punish)
	Intrusiveness	If university can directly track aspects of your well-being, this may lead to disclosing certain information with the university that individuals may not be comfortable with
	Hidden intentions	If the intention of the university would be to use the collected information to improve the general well-being of the overall campus population, then disclosure of information about individual’s well-being would not be needed

### Wearable devices

The first questions were about the familiarity with and preferences for wearable devices. The results showed that smaller devices with minimal designs were favoured. The most favourable being inconspicuous devices such as rings that “you would be wearing anyway” and do not hinder one’s ability to carry out day to day activities. However, the option of a small under the skin or implantable device was also suggested, such as the contraceptive implants or continuous glucose monitoring devices.

Participants expressed comfortability with a device that can function “without you thinking about it”, but that provided useful results. The same propensity for “the less effort it takes, the better” was also echoed here.

The accuracy of the data collected and, thus, the feedback provided was also a consideration for the type of device chosen, a less accurate but inconspicuous device might be preferred for day-to-day activities, but a more accurate and somewhat “unfashionable” or “bulkier” device might be preferred for within the home, whilst asleep, or at certain times when specific measurement is needed.

There was a resoundingly positive response on the reliance on and acceptability of wearables. Participants also expressed comfortability with the pairing of devices or other applications that tracks well-being with those of other users if better results could be obtained. Participants also mentioned that being linked with other peers could give them a sense of reciprocal well-being care.

**Figure 9:**
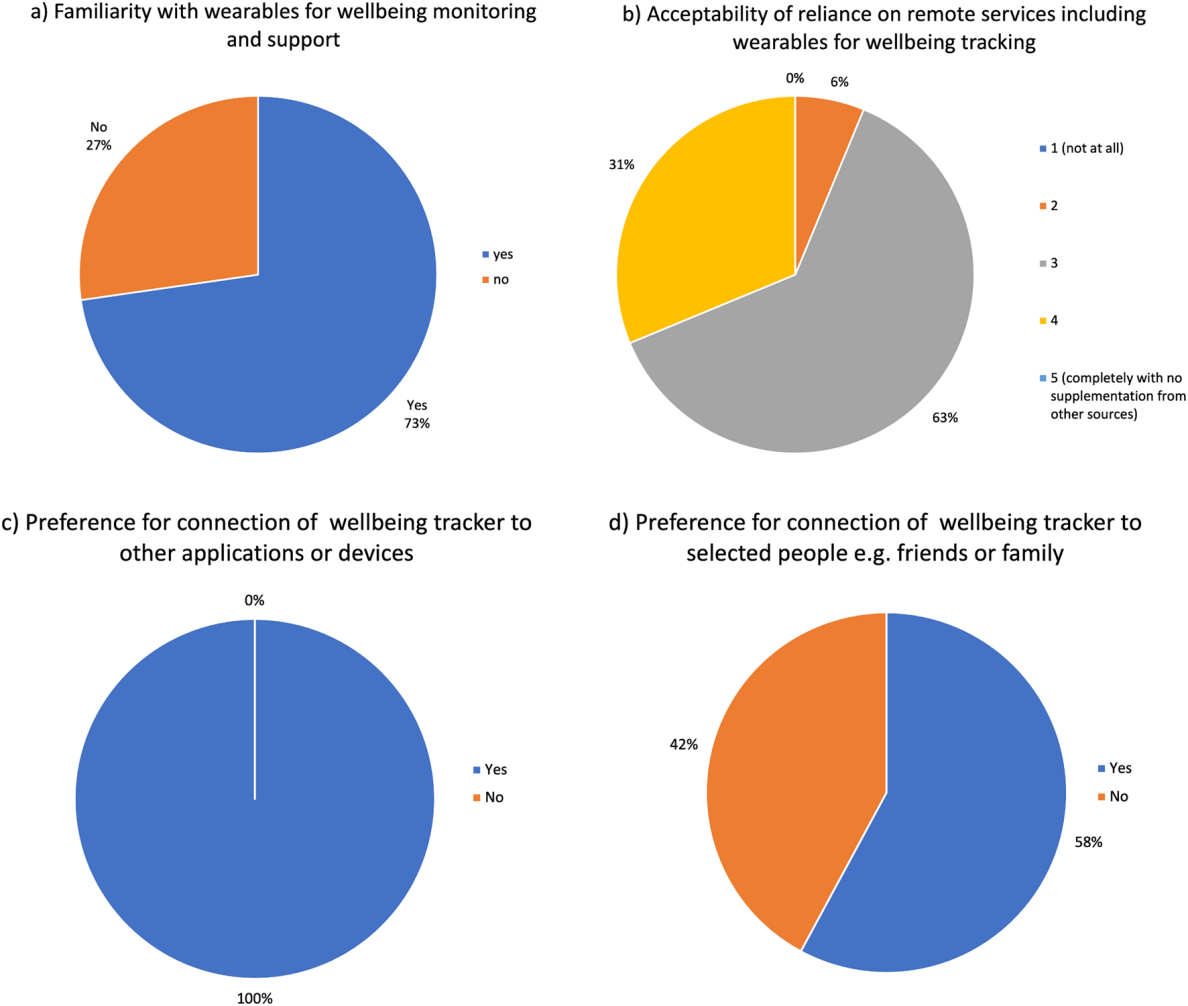
Pie chart representation of poll results of interview questions delivered during the focus group in the sixth section. Questions asked: (**a**) Are you familiar with wearables such as wristband/watch and chest patch for monitoring well-being. (**b**) How much do you think you would rely on wearables to track your well-being? (Likert scale). (**c**) Would you like the device application to connect with other applications or devices? (**d**) Would you like the device to be able to select people e.g., friends or family?

The sixth section of the focus group also included the question on the desired measurable outcome of monitoring and tracking one’s well-being. The main measurable outcomes people seemed to be looking for were related to increased happiness, reduced stress and anxiety, improved concentration, and physical fitness. As most of these rely on intangibles, the best measurable outcome could be using user inputs based on the individual’s perception of these facets (see Fig. 9).

Therefore, a suggested method for presenting a measurable outcome to the user could be through reports generated across a time scale, of the user-input scores or AI suggested values, for all or selected modalities.

## Discussion

The session began similarly to the approach of Powell et al., where their group interview started off with a question about the conceptualization of the word well-being and led to the recognition of the centrality of relationships and sense of agency to enhance well-being^24^. Asking participants “what words come to mind when you think of well-being?” allow them to draw boundaries of their concept of well-being and is key in determining what will be termed and referred to as “individual well-being currency”. An individual’s well-being currency is a quantity used by individuals to measure their well-being. The impressive result related to the frequency of participants thinking to the well-being allowed us to infer that higher-education students are self-conscious and mature enough to make link between well-being and their academic performance, (shown in Fig. 3). Such results were also demonstrated in the Ansari et al.’s findings^16^ and Golsteyn et al.’s one^25^. During the group discussion, most participants expressed their lack of competence in “know how” of well-being tracking. Moreover, they highlighted that even if they were to receive training, it would still be difficult to manage and very time-consuming.

Therefore, the conscious awareness is quickly postponed due to the direct urgency of everyday life tasks. Although well-being is determined to be important and useful, the lack of time appears to be the true and sometimes unconscious limitation to tracking it. This strongly highlights the importance of developing a device or service for monitoring their well-being in a way that is easier and more sustainable than existing methods.

The second section shows the participant’s opinion on tracking solutions for well-being monitoring. The interview revealed that participants were only motivated to track their well-being when something upsetting happens, reactively, to get things under control or discover the source of the disturbance. Instead of this, a more useful solution, which they acknowledged, would be a continuous monitoring system/service that could help prevent negative events or early detect the warning signs. The discussion revealed that for participants to find this manageable, automation of the tracking process would be needed. In general, participants were puzzled by excessive interaction with the app, either for entering data or receiving responses, especially the latter. Indeed, they reported that receiving responses is critical, as it can be a major source of extra stress. This highlights how well-being tracking can be a “double-edged sword”. In fact, being more aware of your actions that negatively impact your well-being can be a source of stress. Moreover, tracking well-being can be a time-consuming effort and a source of distraction. Some participants were worried that tracking well-being may detract focus from other current priorities. The discussion revealed that this depended on the amount of needed effort, as most users would like to track their well-being for an extended period of time (as shown in Fig. 4) in a sustainable way. Lastly, possible subliminal persuasion surfaced. Participants noticed that some services for monitoring and supporting well-being can be used to urge individuals to use and try particular products or services for financial gain to the service provider. They would prefer to avoid this as much as possible.

The unquantifiable nature of well-being due to its “multifaceted” nature was a recurring theme in the discussion of the tracking. Although the majority of participants thought that tracking one's well-being would result in the improvement of one's well-being, as shown in Fig. 3 its “complexity” was the main cause of the (mis-)perceived futility of well-being tracking. The evidence does not, however, strongly support this.

In fact, there are readily available tools that can track and measure well-being that are mostly based on physical fitness and sleep tracking devices^26^. Furthermore, future advancement in development of this kind of tools should involve all the mental health and psychological part of the well-being^27^. An important result was the fact that participants were worried that tracking their well-being could become an obsessive activity and a distraction. Very few studies elaborate on this concern, and most of them focus on fitness apps, rather than general well-being and this research path should be explored further and incorporated in the design of a service ^28^. Lack of sufficient motivation and difficult-to-use device were two of the main barriers to well-being apps’ utilisation found by Ahtinen et al.^29^. The discussion's findings corroborated this, indicating that the main obstacle to the participants in this group achieving the desired outcome of improved well-being was a lack of consistency with tracking, primarily because of a perception of futility or lack of manageability. Therefore, these issues should be taken into consideration when designing a better service or tracking device for well-being that users will be happy with.

The concluding remarks from the third section discussion revealed that participants were largely indifferent about the data analysis process to track and provide feedback on their well-being. Although a specialist was preferred, it was not deemed necessary if the received feedback is useful. Nevertheless, relying on a device is something that still raises doubts (inaccuracy, lack of expertise, uncertainty). Many publications and official international documents now debate on the Trustworthy AI^30–33^, however, the idea of a specialized competence available coming from a human (e.g., a doctor or an engineer, a professional in general) guarantees greater reliability of the technology per se^34^. Overall, these can be circumvented by using machine learning techniques trained using data presented to a professional in the field. This could increase the acceptability of such solutions along with the full transparency of how the service works.

Therefore, this approach aimed at supporting employees’ well-being can have a repercussion considering the information collected: there must be in place well defined in the data privacy management plan. Otherwise, there could be negative repercussions for employees. It is well known that in past the misuse of personal/health related information has been used against the employee^35^, and for this a stricter approach is needed. On the other hand, employees may also benefit from this support for their health by requesting time off when it is not actually necessary.

The conclusion from the fourth section, about the data collection and receival supports the joining of different methods (a combination of user input and automatically derived information).

The results from this section show that the optimal well-being tracker should rely more on data automatically derived either from sensors or behaviour inferences rather than direct user-input. It was mostly stressed that the data collection and input should require minimal attention and effort from the user. The discussion showed that a threshold above (i.e., once input per day) would no longer be acceptable for user-input entry for the data, and the gaps should be filled using automatic data collection, i.e., with the use of step counting, heart rate data, etc. For instance, only one user interaction per day should be allowed for inputs related to mood or eating habits. This was decided upon to allay worries about obsessive tracking that could develop due to how frequently input is gathered.

It is important to acknowledge the concerns raised about the potential for incorrect inferences about well-being states to be drawn from automatically generated data from continuous measurements. To maximise the optimization of their well-being currency, users should be able to adjust how much of the data used for the various features measured is automatically derived versus user-input. Additionally, whenever practical, inferences drawn from the data should be periodically double-checked with the user. The comparison between user input and derived information was a final insightful point made. This can be used as a resource for information about one's health and is particularly helpful for people with affective disorders^36^.

The fifth section was about tracking of well-being by the University. Although UoW has in place a well-developed system for supporting the well-being of students and staff, many suggestions were proposed for improvement. Overall, a solution that makes well-being tracking by the University more acceptable requires transparency of the University about what information the well-being tracking service will disclose to the University. For an even better service, individuals should be able to selection options for what information they are willing to disclose with strict policies enforcing adherence with their choices. An overall consideration is to spread this kind of approach to all universities, the academic institution and in general all the workplaces^37,38^.

The sixth section goes in a way slightly different: although previously participants voiced doubts in relying on technology, in this section they had preference of very automatized process of tracking, that should not conflict with their own life. This corresponds with the removal of the unwanted negatives of obsessive tracking, or a source of stress and the propensity for “the less effort it takes, the better”, a point previously raised in section “Methods”. However, the interesting result is that participants do not care about invasiveness of the devices in their own body but to changes in their habits. From this it possible to infer that there is resistance to improve their well-being if this affects their socio-environmental relationship and requires conceptual redefinition^39^. The accuracy of the data gathered, and the feedback given were major factors in device selection. For daily activities, a less precise but less noticeable device might be preferred. However, a more precise and somewhat "unfavourable" or "bulkier" device might be preferred for use at home, while sleeping, or at specific times when precise measurement is required.

Participants also mentioned that the concept of connecting with others through the tracker can serve as a reminder to assess their own and others' well-being. This could be used to provide a secondary level of well-being awareness, but caution must be adopted to prevent adopting aspects of social media, which are known to have a detrimental impact on well-being.

In the reported six sections, many issues related to ethics emerged *(beneficence principle,* e.g., in the consciousness of the importance of well-being and improving it through tracking, *justice principle* e.g., from the social function of professions and societal expectations of human activities to the cost of specific devices exceeding what an average person could afford, *privacy* and *data protection*, etc.). The well-being is a state of overall mental and physical health, strength, resilience, and fitness to function well at work and personally, different from happiness that is a transient, short-term emotion which cannot be sustained for long^40^. Exaggerated workload and use of social media can lead to numerous physical and psychosomatic disorders^41^. The importance of the use of modern KETs has impacted the holistic well-being on the current generation in terms of enabling for leisure activities such as adherence to routine physical activity^42^. The perception of one’s own well-being seems, therefore, to be one of the priorities in young people. Nevertheless, despite their predisposition, as digital natives, and their interest in wearable technologies, it seems that the basic questions that have been driving the ethical and bioethical debate for years are still causing doubts and uncertainties. Question as data management, data privacy, Trustworthy AI are well debated and, in addition, there is also a lot of regulation on the subject. This somehow should give a sense of security to the users.

The fact that users still have these concerns shows how the debate remains confined to the circle of experts and that the population/users have not yet benefited from the theoretical advances that have occurred (e.g., fear and/or misinformation of users vs certitude of science secure and regulated). This is the real dilemma that represents the limit to the massive spread of tools that could significantly improve people’s well-being, their attitude towards work and study and, therefore, more generally the quality of life.

Another key result is the reframing of the intrusion concept. It seems that experts, to make the technologies as acceptable as possible, tend to design solutions as less intrusive as possible (normally not invasive). In addition, to make technologies acceptable, designers always try to encourage interactive design solutions, in which the user collaborates in the data collection process, also in order not to give the impression that something can escape their control. On the other hand, it appears from the focus groups that users prefer fully automated mechanisms with little need for interaction. They also seem to fully accept the possible invasiveness (instrusivity) of the devices, to the point where even a physical intrusion into the user's body is preferred to user interaction. All of this, so long as the intrusion does not interfere with routine motions or activities. This result must likely be viewed in light of the aforementioned: the viewpoint of young people who are engineering students and are familiar with technologies. However, this does not rule out the possibility that users from other categories may also hold the same opinion.

In conclusion a deep interconnection between ethics and biomedical engineering emerged, both because it is essential for modern health technology design and because the loose concept of well-being represents itself the ideal topic for interdisciplinary researchers^43,44^.

## Conclusion

Well-being promotion on campus was one of the starting points of this study, ensuring a healthy environment for both students and staff led to the collaboration between the Warwick team and BT. This study assessed the feasibility of KETs for well-being monitoring while evaluating the user's perception and acceptability, conducting a focus group on the topic.

This paper provides useful insight into people’s expectations on technological devices intended for monitoring and promoting well-being. This knowledge can be beneficial in the designing process of new technologies, aiming to support well-being; helping not only to think deeper into proper well-being and its importance for quality of life but even to shape and successfully adopt more effective and accepted devices.

The participants are representative of real users, particularly younger generations. Some of them will be future biomedical engineers: this enhances the importance and utility of this study, allowing to shape solutions according to their needs and technical suggestions. We found that most of the participants were willing to track their well-being through a technological device, even invasive i.e., preferring the benefit of the measure of data to enhance well-being compared to the fear of it taking control. However, participants found themselves to be lacking in the knowledge on how to properly track their well-being, and concerns on the multi-faced nature of well-being were raised. At the same time, participants shown to be interested in well-being monitoring technologies, if an easy-to-use and an automated system is in place.

It is important to highlight that a flexible and reliable data collection method is vital to the usefulness of these devices, and minimal data input for the user could help with accessibility. Therefore, a semi-automated (mainly automated) method of data input would be optimal. This is because participants seem to prefer automated solutions with minimal to no requirements and tasks for the users.

The combination of both human and artificial intelligence methods for data assessment could represent an innovative strategy in this regard. Monitoring well-being should not be the only goal; well-being improvement should be pursued as a goal. Finally, it is critical to stress how crucial it is to give inclusion and diversity consideration during the development process.

Ethics has a strong role in the questions posed during the focus group and the most interesting ethical dilemma registered is a bidirectional hiatus one. The first is between experts and users because it seems that many users' doubts or concerns are really outdated and overcome by experts: a better dialogue and a specific sensibilization on the topic of well-being would favour a better acceptance of such technologies. The second hiatus is on the other way round: between users and experts. The users, in fact seem to accept technologies as automated as possible and even intrusive, which experts tend to avoid. This, again, shows the need of dialogue among those categories that could be easily conducted through focus groups as the one here presented.

The relevance of ethical issues emerging here reinforced the idea that ethics and engineering are deeply interconnected, even more if in the field of healthcare. Well-being can be a point of connection to be addressed under an interdisciplinary lens, to improve the quality of life of technology users (including university students and staff).

### Supplementary Information

Supplementary Information 1.

Supplementary Information 2.

## Data Availability

The datasets used and/or analysed during the current study are available from the corresponding author on reasonable request.
