# Supplementary material for: Ethics and biomedical engineering for well-being: a cocreation study of remote services for monitoring and support
Source: Sci Rep. 2023 Aug 31;13:14322. doi: 10.1038/s41598-023-39834-8 (PMC10471689; doi:10.1038/s41598-023-39834-8)
Supplement: Supplementary file 1 — Supplementary Information 1. [file 41598_2023_39834_MOESM1_ESM.docx]

# Supplementary material 2

## Focus Group 1

### The Concept of and Thoughts of Wellbeing

- Wellbeing tracking behaviour – Do you currently track your wellbeing?
- Frequency of thoughts concerning one’s wellbeing - How often do you think about your wellbeing?

- Thoughts on effect of tracking on improvement of wellbeing – Do you think that monitoring your level of well-being can allow you to improve it?
- Importance of wellbeing tracking – How important is it for you to be able to track and improve your wellbeing?

### University and your wellbeing

- Perception of University’s consideration of staff and students’ wellbeing - Do you think that the University cares about your wellbeing?
- Thoughts on University using remote wellbeing services – Would you be comfortable with the university tracking your wellbeing using remote services?

### Wellbeing tracking: Data collection, methods, frequency, and analysis

- Method of data entry into remote wellbeing service– Would you prefer to the wellbeing tracker to reply on user-input or automatically derived (e.g. using sensors)?
- Form of data collection – How would you like the information about your wellbeing to be collected?
- Frequency of data collection – How often would you like the required information should be tracked and collected?

### Evaluating Wellbeing monitoring and support services

- Assumed usefulness of remote wellbeing services – Do you think remote services for monitoring and support wellbeing are useful?^[[1]](#footnote-2)^
- Confidence with the use of AI to track wellbeing – How comfortable are you with the use of artificial intelligence to monitor your wellbeing?

### Wearable Devices

- Preference for wellbeing app connected to other applications or devices – Would you like the wellbeing tracker device to be able to couple to other types of devices or applications?^[[2]](#footnote-3)^

### Wellbeing tracking; duration, reasons, and reservations

- Preferred duration for wellbeing tracking – Over how long would you be interested in tracking your wellbeing?

### Word Cloud Responses:

Word cloud representation of written responses for the first focus group cohort.

Note: Word size reflects the frequency in the responses provided by the participants, orientation and distance between words are random.

-
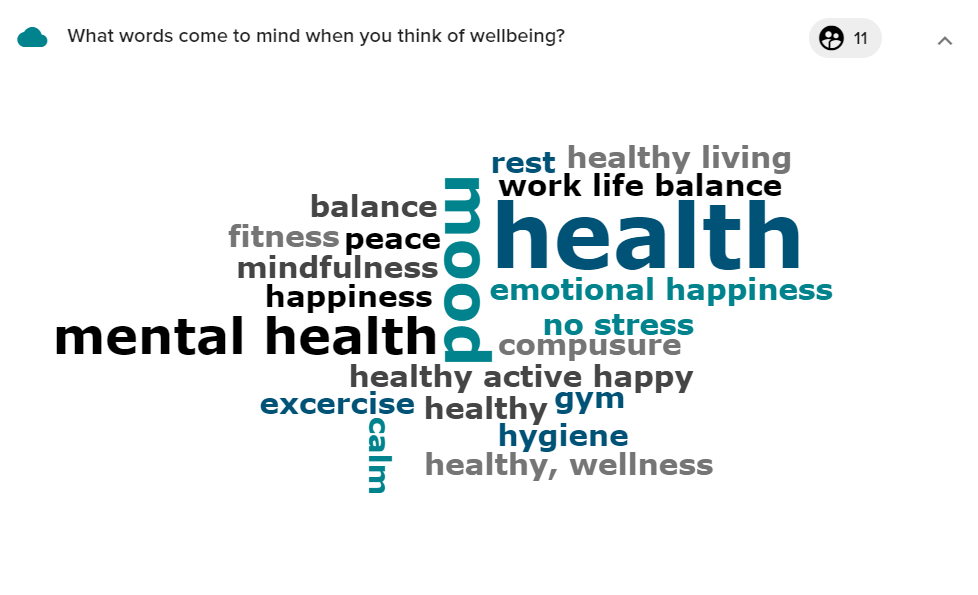
What words come to mind when you think of wellbeing?
-
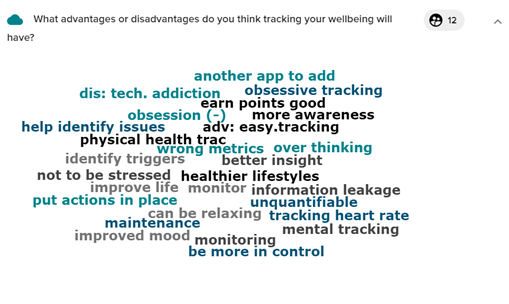
What advantages and disadvantages do you think tracking your wellbeing will have?
- What kind of information, physiological or otherwise, do you think can provide information about one’s wellbeing?


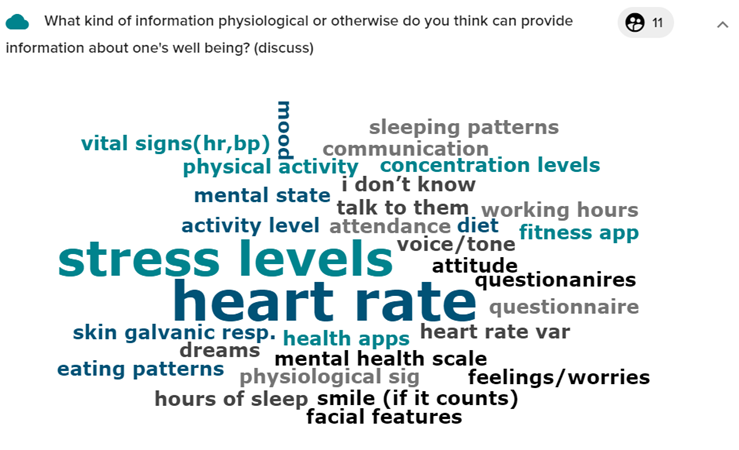


- What kind of measurable outcome would you like to benefit from a wellbeing monitoring device?


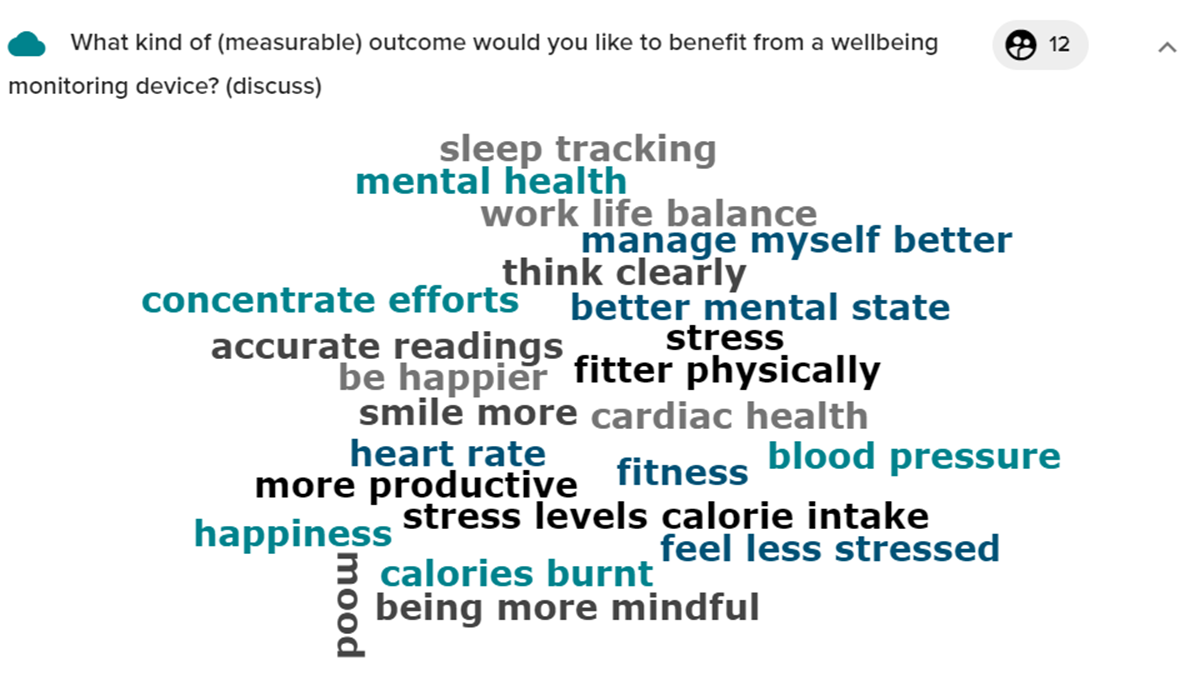


## Focus Group 2

### The Concept of and Thoughts of Wellbeing

- Wellbeing tracking behaviour – Do you currently track your wellbeing?

- Frequency of thoughts concerning one’s wellbeing - How often do you think about your wellbeing?
- Thoughts on effect of tracking on improvement of wellbeing – Do you think that monitoring your level of well-being can allow you to improve it?
- Importance of wellbeing tracking – How important is it for you to be able to track and improve your wellbeing?^[[3]](#footnote-4)^

### University and your wellbeing

- Perception of University’s consideration of staff and students’ wellbeing - Do you think that the University cares about your wellbeing?
- Thoughts on University using remote wellbeing services – Would you be comfortable with the university tracking your wellbeing using remote services?

### Wellbeing tracking: Data collection, methods, frequency, and analysis

- Method of data entry into remote wellbeing service– Would you prefer to the wellbeing tracker to reply on user-input or automatically derived (e.g using sensors)?^[[4]](#footnote-5)^
- Method of receiving suggestions and data collection – How would you like to receive the suggestions of implementations to improve your wellbeing and like your information to be collected?

- Frequency of data collection – How often would you like the required information should be tracked and collected?^[[5]](#footnote-6)^

### Evaluating Wellbeing monitoring and support services

- Assumed usefulness of remote wellbeing services – Do you think remote services for monitoring and support wellbeing are useful?
- Confidence with the use of AI to track wellbeing – How comfortable are you with the use of artificial intelligence to monitor your wellbeing?

### Wearable Devices

- Preference for wellbeing app connected to other applications or devices – Would you like the wellbeing tracker device to be able to couple to other types of devices or applications?

### Wellbeing tracking; duration, reasons, and reservations

- Preferred duration for wellbeing tracking – Over how long would you be interested in tracking your wellbeing?^[[6]](#footnote-7)^

### Word Cloud Responses:

Word cloud representation of written responses for the second focus group cohort.

Note: Word size reflects the frequency in the responses provided by the participants, orientation and distance between words are random.

- What words come to mind when you think of wellbeing?


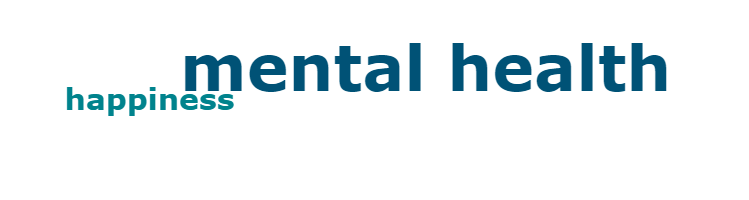


- what advantages and disadvantages do you think tracking your wellbeing will have?


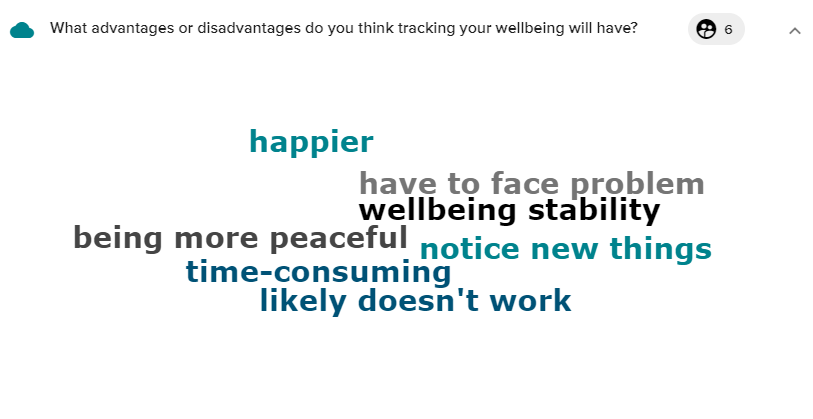


- What kind of information, physiological or otherwise, do you think can provide information about one’s wellbeing?


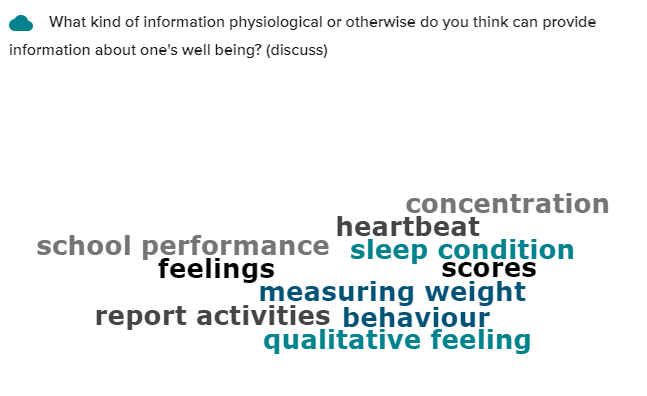


- What kind of measurable outcome would you like to benefit from a wellbeing monitoring device?


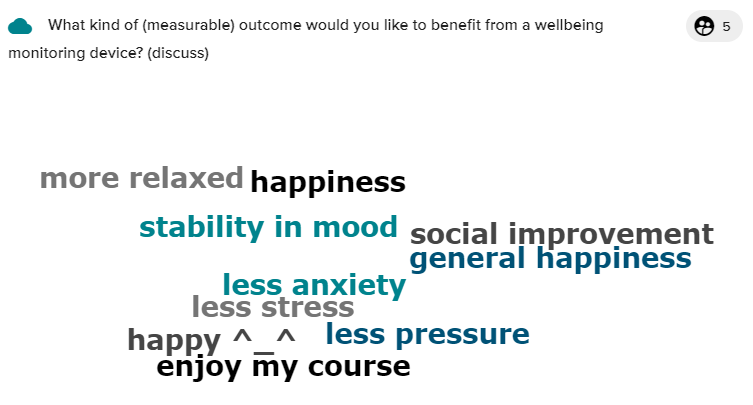


1. This question was added only in the second focus group. [↑](#footnote-ref-2)
2. This question was added only in the second focus group. [↑](#footnote-ref-3)
3. This question was only part of the first focus group. [↑](#footnote-ref-4)
4. This question was only part of the first focus group. [↑](#footnote-ref-5)
5. This question was only part of the first focus group. [↑](#footnote-ref-6)
6. This question was only part of the first focus group. [↑](#footnote-ref-7)
