# Supplementary material for: Ethics and biomedical engineering for well-being: a cocreation study of remote services for monitoring and support
Source: Sci Rep. 2023 Aug 31;13:14322. doi: 10.1038/s41598-023-39834-8 (PMC10471689; doi:10.1038/s41598-023-39834-8)
Supplement: Supplementary file 2 — Supplementary Information 2. [file 41598_2023_39834_MOESM2_ESM.pdf]

Participant Information Leaflet

Study Title: Co-creation study for informing the design of a remote service for wellbeing monitoring and support

Investigator(s): Leandro Pecchia (University of Warwick)

David Wrout, Darren Farmer, Mattew Miller, Claudia Cristina, Simon Beddus, Brian Jackson (British Telecom, BT)

Introduction

You are invited to take part in a research study. Before you decide, you need to understand why the research is being done and what it would involve for you. Please take the time to read the following information carefully. Talk to others about the study if you wish.

Please ask us if there is anything that is not clear or if you would like more information. Take time to decide whether or not you wish to take part.

Who is organising and funding the study?

The study is organised by the Applied Biomedical Signal Processing Intelligent eHealth Lab of the University of Warwick, in collaboration with British Telecom (BT), which is sponsoring this co-creation study.

What is the study about?

The study is about the wellbeing of university students and staff, and the possibility of monitoring and supporting it with the help of Artificial Intelligence (AI) and Internet of Things (IoT). This is a preliminary study, aimed at collecting information for the design of a research on the remote monitoring and support of wellbeing with the use of AI and IoT. In addition, we can consider this a co-creation study, i.e., a study structured together with the users. To this purpose, you (the user), will help design the study and assess the feasibility of the future research stemming from this.

For this reason, this preliminary co-creation study will be focused on understanding your (either staff or student) idea of wellbeing, as well as your needs, the identification of potential risks and effective mitigation strategies. Related to this, a part of the study will focus on the investigation of your acceptability of AI and IoT for monitoring your wellbeing. During the focus group, your expectations from a remote service for monitoring and support wellbeing will also be deepened. This information will help the future study for assessing the use of remote services for wellbeing monitoring and support.

A research group will be at your complete disposal to support and guide you, throughout the study.

What would taking part involve?

You can be involved, if you are a student or a staff member of the Warwick University.
If you accept to participate in this study, before taking part, you will be briefed about it together with other students/staff in a meeting via Teams.

After this, you and other participants will be invited to join the co-creation group meeting, during which the moderator will ask you and the other participants to sign a consent form online. Upon obtaining the consent, the moderator will explain the purpose of the focus group, the ground rules, and the goals of the meeting, while encouraging open participation. The focus group will be audio recorded via the Microsoft Teams built-in recorder.

After the introduction, the moderator will start the open-floor discussion.

Different tools and techniques (questionnaires, surveys, brainstorming, etc.), could be used during the meeting, and their results will be collected at the end with your and otherparticipants' feedback.

The moderator will be supported by their research team to take notes. The whole meeting would last approximately 90-120 minutes.

All the acquired data will be pseudonymised and encrypted. This means that you will be assigned a participant identification (PID) number by the project PI (Prof Pecchia). Only Prof Pecchia will know which participant corresponds to each PID, and this information will be stored in a secure manner using an encrypted file.
All the collected data will be pseudonymized and encrypted and it will not be possible to identify any of the participants from the stored information.
All data shared with BT will be completely anonymised as far as BT is concerned, in fact it won't be possible to associate data with an identifiable individual.

Do I have to take part?

No. Participation in this study is completely voluntary and choosing not to take part will not affect you in any way. You can also choose to withdraw your participation at any time, without giving a reason by contacting one of the research team. Further details about withdrawing from the study are provided later in this document.

What are the possible benefits of taking part in this study?

Although there are no direct benefits for you, your participation is particularly useful to co-create the study design of a project related to test technologies that can be combined for designing a solution for wellbeing monitoring. These results can also inform future project proposals exploring if the same technology can be used for the development of risk management systems.

What are the possible disadvantages, side effects or risks, of taking part in this study?

No disadvantages, significant side effects, risks, and/or discomforts were registered during

similar studies.
You might feel anxious when sharing your opinion and you may be worried about the potential disclosure of personal information. Please notice that the moderator will make sure that you and the other participants feel at ease and in a protected environment, in which you can freely share your opinion.
Data collected from interviews will be pseudonymized.

Expenses and payments

Participation in the study will not cost you anything and no payments will be made for participation.

Will my taking part be kept confidential?

All the acquired data will be pseudo-anonymised. This means that you will be assigned a participant identification (PID) number by the project PI (Prof Pecchia). Only Prof Pecchia will know which participant correspond to each PID, and this information will be stored in a secure manner using an encrypted file on a password protected computer. The pseudo-anonymised research data will be stored at the University of Warwick for 10 years.
The results from the study will be stored and eventually published on journal/conference scientific papers.

What will happen to the data collected about me?

As a publicly funded organisation, the University of Warwick have to ensure that it is in the public interest when we use personally identifiable information from people who have agreed to take part in research. This means that when you agree to take part in a research study, such as this, we will use your data in the ways needed to conduct and analyse the research study.

We will be using information from you in order to undertake this study and will act as the data controller for this study. We are committed to protecting the rights of individuals in line with data protection legislation. The record of the focus group will be deleted as soon as possible after their transcription, while the anonymised transcripts will be kept in a safe and encrypted storage and will be deleted after 10 years from the study taking place

Research data will be **pseudonymised** as quickly as possible after data collection. This means all direct and indirect identifiers will be removed from the research data and will be replaced with a participant number. The key to identification will be stored separately and securely to the research data to safeguard your identity.

If you wish to withdraw your data, it will be possible up to 1 week after the experiment taking place, by contacting the main investigators.

Data Sharing

Your rights to access, change or move your information are limited, as we need to manage your information in specific ways in order for the research to be reliable and accurate. The University of Warwick has in place policies and procedures to keep your data safe.

This data may also be used for future research, including impact activities following review and approval by an independent Research Ethics Committee and subject to your consent at the outset of this research project.

For further information, please refer to the University of Warwick Research Privacy Notice which is available here: https://warwick.ac.uk/services/idc/dataprotection/privacynotices/researchprivacynotice or by contacting the Information and Data Compliance Team at GDPR@warwick.ac.uk.

British Telecom will receive your data completely anonymized. This data wouldn't be associated with any identifiable subject.
British Telecom has in place policies and procedures to keep your data safe.

Lawful basis

This project is in line with the UK GDPR for data processing of personal data handled as part of the research study. In line with the Article 6 of the UK GDPR, the lawful bases for processing personal data in this research study are:

a) Consent: pseudonymized data (feedback)
b) Legitimate interest: age, gender, email.

What will happen if I don't want to carry on being part of the study?

Participation in this study is entirely voluntary. Refusal to participate will not affect you in any way, and that you are free to withdraw at any time without giving any reason, without your *education, or legal rights* being affected. If you decide to take part in the study, by signing the consent form, you are allowing the use of the provided data for the research project. During any time of the experiment, you can decide to stop taking part to the study without any problem. You can also decide to withdraw your data from the study, by contacting the investigators up to 1 week after your experiment session took place.

To safeguard your rights, we will use the minimum personally identifiable information possible and keep the data secure in line with the University's Information and Data Compliance policies.

What will happen to the results of the study?

The data resulting from the study will be used to draft the study design of the BT-UoW project. If this feasibility pilot study will be successful, the Warwick and the BT teams will work together for writing a more ambitious project leading to the designing and testing of a system for wellbeing monitoring.

Who has reviewed the study?
This study has been reviewed and given favourable opinion by the University of Warwick's Biomedical & Scientific Research Ethics Committee (BSREC)

Who should I contact if I want further information?
If you have any questions about any aspect of the study, or your participation in it, not answered by this participant information leaflet, please contact:

Leandro Pecchia, L.Pecchia@warwick.ac.uk

Who should I contact if I wish to make a complaint?

Any complaint about the way you have been dealt with during the study or any possible harm you might have suffered will be addressed. Please address your complaint to the person below, who is a senior University of Warwick official entirely independent of this study:

Head of Research Governance
Research & Impact Services
University House
University of Warwick
Coventry
CV4 8UW
Email: researchgovernance@warwick.ac.uk
Tel: 02476 575733

If you wish to raise a complaint on how we have handled your personal data, you can contact our Data Protection Officer who will investigate the matter: DPO@warwick.ac.uk.

If you are not satisfied with our response or believe we are processing your personal data in a way that is not lawful you can complain to the Information Commissioner's Office (ICO).

Thank you for taking the time to read this Participant Information Leaflet
